# Predicting PDZ domain mediated protein interactions from structure

**DOI:** 10.1186/1471-2105-14-27

**Published:** 2013-01-21

**Authors:** Shirley Hui, Xiang Xing, Gary D Bader

**Affiliations:** 1 The Donnelly Centre, University of Toronto, Toronto, ON, Canada; 2 Department of Molecular Genetics, University of Toronto, Toronto, ON, Canada; 3 Department of Computer Science, University of Toronto, Toronto, ON, Canada

## Abstract

**Background:**

PDZ domains are structural protein domains that recognize simple linear amino acid motifs, often at protein C-termini, and mediate protein-protein interactions (PPIs) in important biological processes, such as ion channel regulation, cell polarity and neural development. PDZ domain-peptide interaction predictors have been developed based on domain and peptide sequence information. Since domain structure is known to influence binding specificity, we hypothesized that structural information could be used to predict new interactions compared to sequence-based predictors.

**Results:**

We developed a novel computational predictor of PDZ domain and C-terminal peptide interactions using a support vector machine trained with PDZ domain structure and peptide sequence information. Performance was estimated using extensive cross validation testing. We used the structure-based predictor to scan the human proteome for ligands of 218 PDZ domains and show that the predictions correspond to known PDZ domain-peptide interactions and PPIs in curated databases. The structure-based predictor is complementary to the sequence-based predictor, finding unique known and novel PPIs, and is less dependent on training–testing domain sequence similarity. We used a functional enrichment analysis of our hits to create a predicted map of PDZ domain biology. This map highlights PDZ domain involvement in diverse biological processes, some only found by the structure-based predictor. Based on this analysis, we predict novel PDZ domain involvement in xenobiotic metabolism and suggest new interactions for other processes including wound healing and Wnt signalling.

**Conclusions:**

We built a structure-based predictor of PDZ domain-peptide interactions, which can be used to scan C-terminal proteomes for PDZ interactions. We also show that the structure-based predictor finds many known PDZ mediated PPIs in human that were not found by our previous sequence-based predictor and is less dependent on training–testing domain sequence similarity. Using both predictors, we defined a functional map of human PDZ domain biology and predict novel PDZ domain function. Users may access our structure-based and previous sequence-based predictors at http://webservice.baderlab.org/domains/POW.

## Background

**Figure 1:**
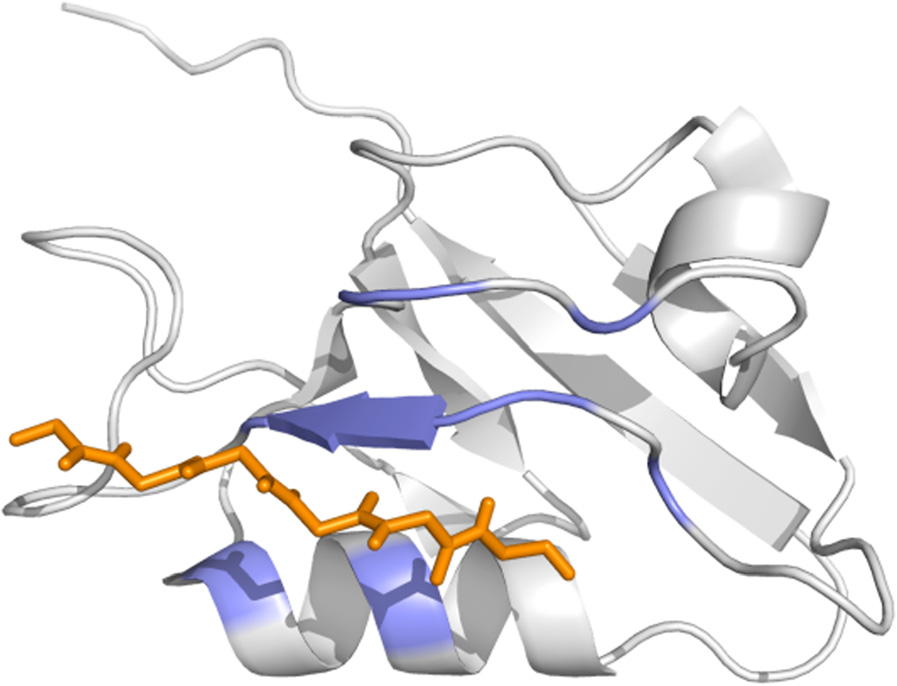
**3D structure of a bound PDZ domain.** The PDZ domain folds into a structure consisting of six β strands and two α helices. Canonical interactions occur through C-terminal target side chain interactions and the hydrophobic domain binding pocket formed between domain strand β2 and helix α2. The ten core domain binding sites are highlighted in blue and the bound peptide (RRETQV) is in orange. PDB:2OQS (NMR first model) [74].

**P**SD95/**D**lgA/**Z**o-1 (PDZ) domains are modular peptide recognition domains that are generally found in eukaryotic signalling pathways, often in scaffolding proteins that are responsible for regulating protein complex assembly and localization to specialized sites in the cell, especially at membranes [1]. Their importance in higher organisms is highlighted by their increasing abundance from yeast to human (with only 2 in yeast and over 250 encoded in the human genome) and association with diseases such as cystic fibrosis and schizophrenia, and pathogens, such as human papillomavirus [2-4]. PDZ domains fold into a globular structure consisting of six β strands and two α helices (Figure 1) and often bind their targets through the recognition of hydrophobic C-termini. Canonical interactions occur between the target peptide side chains and a hydrophobic binding pocket formed between domain β2 strand and α2 helix, though other binding modes are known. The binding specificity of PDZ domains has been categorized into two main classes, where class I domains prefer to bind C-terminal motifs X[S/T]XΦ and class II domains prefer to bind XΦXΦ (where X is any amino acid and Φ is a hydrophobe) [5]. More recent studies have found that the PDZ domain can be specific up to seven residues [6,7].

Recent high throughput experiments have resulted in the availability of large data sets of PDZ domain-peptide interactions [7,8]. As a result, several computational methods have been developed to predict PDZ domain-peptide interactions using sequence-based information only [8-12]. Previously, we developed a sequence-based predictor to scan proteomes of multiple organisms for binders of PDZ domains [10]. Although this predictor is more accurate and precise at proteome scanning compared to previous sequence-based predictors, like others, it performs better on sequences similar to those in the training set. It is known that structure features within the domain binding pocket play important roles in determining binding specificity [13-15]. Since domain structure features capture different information about binding compared to sequence features, we hypothesized that training with such features would result in a predictor that is complementary to the sequence-based predictor. In particular, such a predictor would be less dependent on sequence similarity and would predict additional interactions not predicted by the sequence-based predictor. This would expand the coverage of PDZ domain C-terminal peptide interactions that can currently be predicted by sequence-based predictors alone.

Structure-based predictors have been developed to more generally predict protein-protein interactions. For instance, Hue et al., used a support vector machine (SVM) to predict PPIs using a structure kernel [16]. Methods utilizing structure information to more specifically predict PPIs mediated by peptide recognition domains have also been developed. Sanchez et al., used an empirical force field to calculate structure-based energy functions for human SH2 domain interactions [17]. Fernandez-Ballester et al., constructed position weight matrices of all possible SH3-ligand complexes in yeast using homology modelling [18]. Smith et al., used protein backbone sampling to predict binding specificity for 85 human PDZ domains [19]. Kaufmann et al., developed an optimized energy function to predict the binding specificity of PDZ domain-peptide interactions for 12 PDZ domains [20].

In this paper, we present a structure-based predictor for PDZ domain-peptide interactions that can be used for proteome scanning. Our predictor uses a variety of different structure features that are known to play roles in protein structure stability and facilitating PPIs. Through leave 12% of domain out cross validation, we showed that the structure-based predictor depends less on training–testing domain sequence similarity compared to our previous sequence-based predictor. Based on human proteome scanning results, we also show that the structure-based predictions correspond to known experimentally determined PDZ domain-peptide interactions and known PPIs involving PDZ domain containing proteins. A substantial number of the structure-based predictions correspond to known PPIs not previously predicted by the sequence-based predictor (48% increase), confirming that the structure-based predictor finds different interactions than the sequence-based predictor. Using predictions from both methods, we created a functional map using all predicted human PDZ mediated PPIs and identify xenobiotic metabolism as a novel biological process enriched in PDZ interactors.

Finally, we developed a website called POW! PDZ domain-peptide interaction prediction website (http://webservice.baderlab.org/domains/POW), which enables users to run our sequence-based and structure-based predictors online in human, mouse, fly and worm.

## Methods

### Domain binding site definition

A number of positions in the PDZ domain that are in close contact with the peptide are important for binding [7,8]. Following previous work, we defined the binding site using ten domain positions (core positions) that are in close contact with the peptide ligand (< 4.5 angstroms) across nine PDZ domain structures. In total, 218 out of 267 human PDZ domains could be used because they didn’t have gaps in their binding sites based on a PDZ family multiple sequence alignment (8 structures), and we could obtain structures and compute features for them (41 structures). For mouse, fly and worm, respectively, 178 of 237, 85 of 117 and 64 of 81 known PDZ domains were supported with 11, 14 and 7 of the remaining domains containing gaps. All PDZ domains were defined by HMMER 3.0 [21] against UniProt defined PDZ proteins as of Apr 2012. Overall, the structure-based predictor supports the majority of PDZ domains (i.e. 82%, 74%, 73% and 79% of known PDZ domains) for human, mouse, fly and worm, respectively.

Although previous studies used a binding site definition of 16 domain positions (a superset of the ten we use), these positions were identified from only a single PDZ domain-peptide complex structure [9,10] and many domains contain gaps using this larger 16-position binding site definition (based on a multiple sequence alignment with other PDZ domains). A comparison of cross validation performance (see section on Predictor Performance Evaluation) using ten versus 16 binding site positions showed that the ten positions were adequate for achieving good predictor performance (see Additional file 1: Table S1).

### Domain structure data

The initial set of PDZ domain structures consists of one NMR and 17 X-ray structures for human collected from the Protein Data Bank (PDB) [22] with corresponding interaction data from phage display or protein microarray experiments [7,8]. Five NMR structures were collected from the PDB for mouse. For NMR structures, only the first model was used. Homology models were used to increase the number of structures available for domain structure feature encoding. In total, 11 human and 54 mouse PDZ domain models were modelled by SWISS-MODEL [23] (downloaded Feb-Sep 2011) through the Protein Model Portal, which is a website providing access to structure models generated by different protein structure resources [24].

The quality of the homology models was estimated by computing the number of identical residues between the target and template sequence (i.e. template sequence identity). It has been shown that target-template sequence identity is positively correlated with model quality. In particular, state-of-the-art algorithms can always build high quality models (RMSD < 2 Å) if the target-template sequence identity is higher than 35-40%. Furthermore, there is no significant variation in model quality for targets with sequence similarity between 40-70%. If the similarity is 35%, there is no correlation [25,26]. All training models have greater than 50% sequence similarity to their template structure (average 90%). At this threshold, models are expected to have the correct fold with most inaccuracies arising from structural variation in templates and incorrect reconstruction of loops [25,26]. We also computed the QMEAN score which is a scoring function measuring multiple geometrical aspects of protein structure including torsion angle potential, secondary structure-specific interaction potentials and solvation exposure potential [27]. This score ranges from zero to one with scores closer to one indicating more reliable models. The minimum QMEAN score for our training models is 0.520 (average 0.836). Please see Additional file 2: Table S1 for details on all training domains.

### Domain-peptide interaction data

PDZ domain-peptide interactions were collected from published high throughput phage display and protein microarray experiments for human and mouse, respectively [7,8]. Since the phage display data consisted of only positive interactions (of which many could be non-genomic, meaning not similar to any genomic peptide), we used an established protocol to filter the interactions to enrich for genomic interactions and to generate artificial negative interactions [10]. Briefly, this protocol involves creating a position weight matrix for a given training domain using its experimentally determined binders (positives) and then using the matrix to scan a pool of C-terminal peptides (last 5 positions) for low scoring binders (negatives). We adopted a minor modification of this procedure to allow for the inclusion of additional class II type PDZ domains to increase coverage of the PDZ family – the minimum number of genomic peptides required for inclusion was relaxed from ten to four. Only domains with both positive and negative interaction data were used for predictor training.

### Domain structure feature encoding

Structure features across the entire PDZ domain structure were computed and values corresponding to the ten core binding site positions were extracted from the larger list of features computed for all domain positions. Four types of structure features (detailed below) involved in protein folding and stability were computed to describe the PDZ domain structure (Figure 1). Three-dimensional geometric descriptors were investigated but were not included because they resulted in inferior cross validation performance (see Additional file 1: Figure S1). In total, the PDZ domain structure as defined by the core positions was represented by a vector of length 240 features. Each value in the feature vector was scaled to lie between zero and one. Details regarding software parameters used to compute the following structure features are available in Additional file 1, section A.

### Solvent accessibility, hydrogen bonding and positive phi angle properties

The first feature type consists of five values describing protein structure and were computed using the JOY web server [28]. Solvent accessibility indicates whether the protein surface in the area at the given core residue position is available to interact with ligands. Therefore, the first value indicates whether a given residue is solvent accessible or inaccessible. Patterns of hydrogen bonding are important in forming protein secondary and tertiary structure and are known to be important for canonical C-terminal peptide binding to the PDZ domain. The next three values indicate if there is a residue side chain hydrogen bonded to a main chain amide, carbonyl or another side chain. Finally, since positive main chain phi angles may restrict what types of residues may be accommodated at a given position, the last value indicates if the residue has a positive phi angle. These binary features (i.e. absence is 0, presence is 1) were computed for each core residue position resulting in a binary vector of length 50 (5 features x 10 core positions).

### Solvent accessible area

The second feature type is a single value indicating how much surface (i.e. area) for a core residue is available for binding to a ligand residue. This feature was computed using the SURFV software [29] for each residue resulting in a numeric vector of length 10 (1 feature x 10 core positions).

### Electrostatic potential and hydrophobicity

Protein-protein interactions are facilitated by the electrostatic and hydrophobic complementarity of molecular surfaces. Therefore, the third and fourth feature types describe the electrostatic potential and hydrophobicity along the surface of the domain. At each core residue position, nine values were sampled from the surface resulting in a total of 90 electrostatic and 90 hydrophobicity values (9 features x 10 core positions). These features were generated by the VASCo software [30].

### Peptide sequence feature encoding

Peptides were encoded using a sparse binary vector encoding, as described in previous work [10]. Briefly, each residue in a peptide of length five was represented using a binary vector of length 20 with each bit corresponding to an amino acid type. The vectors were concatenated to form the final feature vector of length 100.

### Support vector machine

We used the support vector machine (SVM) binary machine learning method for our predictor [31,32]. Given interaction training data (x_1_*y*_1_),…,(x_*m*_*y*_*m*_) where *m* is the number of samples, x_i_ is a feature vector for domain d_i_ and peptide p_i_ and *y* is a class label such that *y*_*i*_ = {−1, +1} [33], the SVM assigns a class label of +1 if a given interaction feature vector encodes a positive interaction or −1 otherwise. The decision function is evaluated to assign the binary label:

(1) $$f\left(x\right)=\text{sgn}\left(w\cdot x+b\right)$$

where sgn(0) = +1, otherwise −1. The weight vector w and bias term *b* describe a maximum margin hyperplane (w,*b*) that separates positive and negative training examples. For such a hyperplane:

(2) $$\text{w}=\sum_{i=1}^{m}\alpha_{i}y_{i}x_{i}$$

where the α_*i*_’s are positive real numbers that maximize the following objective function:

(3) $$\begin{array}{l} \;\sum_{i=1}^{m}\alpha_{i}-\frac{1}{2}\sum_{i,j=1}^{m}\alpha_{i}\alpha_{j}y_{i}y_{j}K\;\left(x_{i},x_{j}\right)\\ \text{subject}\;\text{to the constraints}\;0\leq \alpha_{i}\leq C\;\text{for}\;\text{all}\;i=1,\ldots ,m,\\ \text{and}\;\sum_{i=1}^{m}\alpha_{i}y_{i}=0 \end{array}$$

where *K*(x_i_,x_j_) can be thought of as describing the similarity between two feature vectors, and *C* is a cost parameter that penalizes training errors. We used the radial basis function (RBF) kernel, defined as:

(4) $$K\left(x_{i},x_{j}\right)=e^{-\gamma {\left\|x_{i}-x_{j}\right\|}^{2}}$$

A grid search was used to find locally optimal values for γ and *C*[34]. Instead of explicitly balancing the positive and negative training examples, weighted costs were used according to *C*^*+*^ = (n^+^/n^-^) *C*^*-*^, where n^+^ is the number of positive training interactions and n^-^ is the number of negative training interactions. The LibSVM software library was used to build the SVM [35].

### Semi supervised negative training set expansion

An initial predictor was built using the data for 88 PDZ domains described above. A preliminary assessment of the predictor’s proteome scanning performance was performed by scanning the human proteome (defined by genome assembly Ensembl:37.64) for each domain in the training set. This initial predictor returned a large number of hits (1000 or more) for over half of the domains with an average number of predictions returned per domain of over 2000 (see Additional file 1: Figure S2, left boxplot). Since previous phage display experiments detected fewer than a hundred binders per domain among billions of random peptides, the majority of these initial predictions are likely false positives. We surmised that the initial negative training data did not adequately cover the negative proteomic interaction space. Therefore, we used a semi supervised learning approach similar to a method previously used to expand negative training data sets when there are no negatives initially available [36]. This predictor was used to scan the human proteome for interactors of training domains as we did for the initial predictor. We found that adding negatives reduced the number of hits returned per domain. The final predictor was trained using a total of 942 positive and 1843 negative interactions involving 83 PDZ domains and 872 peptides (Table 1). When scanning the human proteome again, the final predictor predicted 1000 or more hits for only five out of 83 training domains (approximately 6% of training domains). The average number of predictions per domain returned by the final predictor was approximately 400 (see Additional file 1: Figure S2, right boxplot). Please see Additional file 1, section E for more details.

**Table 1:** Summary of the training data

		**Domain**		**Interactions**	
**Organism**	**Source**	**# Pos**	**# Neg**	**# Pos**	**# Neg**
Mouse	Protein microarray	58	53	527	1026
Mouse	SVM Negatives	-	24	-	210
Human	Phage Display	25	-	415	-
Human	PWM Negatives	-	25	-	407
Human	SVM Negatives	-	20	-	200
	Totals	83	-	942	1843

### Predictor performance evaluation

We carried out multiple cross validation strategies to provide an estimate of predictor performance. First we performed ten fold cross validation which involves partitioning the training data into ten randomly selected interaction sets, independently holding out each set for testing against a predictor trained using the remainder of the data, and computing average performance across all ten runs. Following previous prediction methods and to better compare our results with previous work, we held out 12% of the domains (to estimate performance dependence on specific sets of domains), 8% of the peptides (to estimate predictor performance dependence on specific sets of peptides) and both 12% of the domains and 8% of the peptides (to estimate predictor performance dependence on specific sets of domains and peptides) and tested on the rest, again repeating this ten times [9]. In general, the training domain features are more similar to each other (average 0.85 using normalized dot product similarity), compared to the peptide features (average 0.13). Thus, we also performed leave 12% of domains out cross validation with training set filtering based on domain sequence similarity and compared the performance of the structure-based predictor to our previously published sequence-based predictor. This involved holding out all data for 12% of domains for testing and training with only remaining domains and their interactions that had sequence similarity less than a given threshold to all testing domains.

We computed the following statistics to measure predictor performance:

• Sensitivity or Recall: TP/(TP + FN)

• Specificity: TN/(TN + FP)

• Precision: TP/(TP + FP)

where TP is the number of true positives, FP is the number of false positives, TN is the number of true negatives, FP is the number of false positives. The overall performance was summarized by computing the area under the receiver operating characteristic (ROC) curves and Precision/Recall (PR) curves [37,38].

### Functional enrichment analysis

A gene function enrichment analysis was performed on the predicted sequence-based and structure-based gene targets using Gene Ontology (GO) biological process terms [39]. The BiNGO (Biological Network Gene Ontology tool) software library [40] was used to determine the enriched terms. The hypergeometric test was used to compute a *p*-value assessing the GO term enrichment for a given set of predicted genes. Multiple testing correction was performed using the Benjamini and Hochberg False Discovery Rate (FDR) correction. GO v1.2 (downloaded Dec 7, 2011) and human GO annotations (downloaded Dec 7, 2011) were used. Only gene-sets with between five and 300 genes were used from the GO ontology (defined by the GMT file dated Dec 6, 2011 and available at http://www.baderlab.org/Data/StructurePDZProteomeScanning). A list of enriched terms (*p*-value < 0.05 and FDR < 0.1) with more than one gene interactor and associated with more than two domains were retained. To better interpret the structure-based and sequence-based enrichment results, we created an enrichment map, a network-based visual representation of enriched terms that groups similar terms and eases identification of functional themes. We used the Enrichment Map Cytoscape plugin software to create the enrichment map [41,42], using the parameters *p*-value < 0.05, FDR Q value < 0.1 and “Jaccard + overlap similarity” cutoff = 0.517.

## Results

### The structure-based predictor achieves high cross validation results

**Figure 2:**
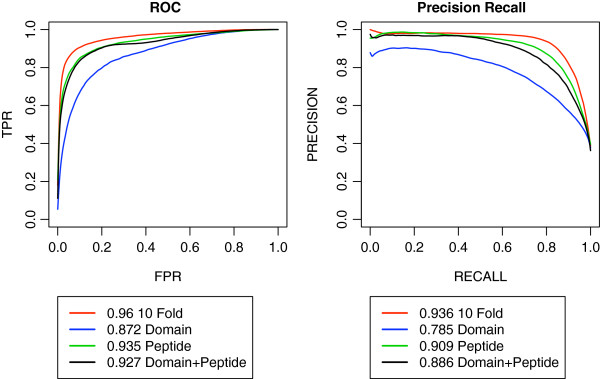
**Predictor performance estimation using cross validation.** Predictor performance measured using ten fold (red), leave 12% of domains out (blue), leave 8% of peptides out (green), leave 12% of domains and 8% of peptides out (black) cross validation.

To estimate the generality of the predictor, we ran multiple cross validation tests and plotted the ROC and PR curves to summarize the performance. The predictor achieves high ROC and PR area under the curve (AUC) scores compared to random performance AUCs over all cross validation strategies. In particular the ten fold cross validation ROC and PR AUCs were 0.96 and 0.936, respectively (random ROC AUC 0.5, PR AUC 0.253). The leave 8% of peptides out cross validation ROC and PR AUCs were 0.935 and 0.909 respectively (random ROC AUC 0.5, PR AUC 0.358). The leave 12% of domains and 8% of peptides out cross validation out ROC and PR AUCs were 0.927 and 0.886 respectively (random ROC AUC 0.5, PR AUC 0.347). Finally, slightly lower AUCs were obtained for the leave 12% of domains out cross validations, which achieved 0.872 and 0.785 respectively (random ROC AUC 0.5, PR AUC 0.33) (Figure 2). Like our previously published sequence-based predictor, the cross validation results were lower for strategies that involved leaving sets of domains out. A one-tailed t-test showed that the mean AUC scores were significantly higher for the structure-based predictor compared to those of the sequence-based predictor (p-value < 0.025) (Table 2). Blind testing results on a small number of genomic mouse, worm and fly interactions suggest that the predictor is able to correctly predict interactions in different organisms. However since these data sets are small, additional data is required to verify this. Please see Additional file 1, section H for blind testing results.

**Table 2:** **Structure-based predictor achieves better cross validation results than the sequence-based predictor (*p*-value < 0.025)**

	**ROC**		**PR**	
	**Structure**	**Sequence**	**Structure**	**Sequence**
10 Fold (95% CI)	**0.96**	0.939	**0.936**	0.896
	(**0.957 ~ 0.962)**	(0.936 ~ 0.941)	**(0.932 ~ 0.940)**	(0.890 ~ 0.900)
Domain (95% CI)	**0.872**	0.851	**0.785**	0.764
	**(0.860 ~0.882)**	(0.839 ~ 0.862)	**(0.765 ~ 0.805)**	(0.747 ~ 0.779)
Peptide (95% CI)	**0.935**	0.893	**0.909**	0.838
	**(0.929 ~ 0.941)**	(0.883 ~ 0.902)	**(0.898 ~ 0.918)**	(0.825 ~ 0.850)
Domain + Peptide (95% CI)	**0.927**	0.87	**0.886**	0.794
	**(0.919 ~ 0.934)**	(0.862 ~ 0.877)	**(0.875 ~ 0.896)**	(0.783 ~ 0.804)

### The structure-based predictor is less dependent on training–testing domain sequence similarity

**Figure 3:**
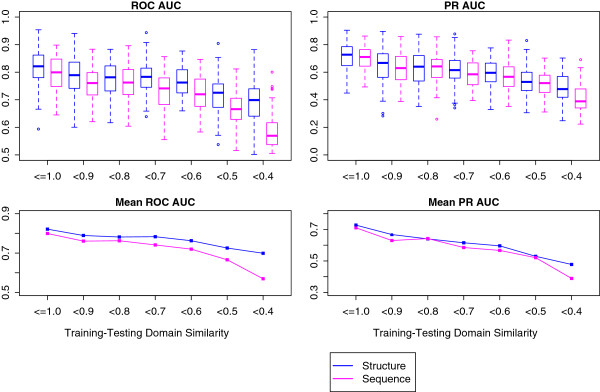
**Predictor performance dependence on training–testing domain sequence similarity.** Leave 12% of domains out cross validation was performed with domains retained for training in each fold if their sequence similarity to all testing domains was less than a given threshold. This was performed for structure-based (blue) and sequence-based predictors (magenta). ROC and PR AUC scores were computed for each run and displayed in box plots according to training–testing domain sequence similarity threshold (top left and right). Based on significance testing using a one-tailed t-test, the mean structure-based predictor ROC and PR AUC scores are significantly higher than the sequence-based predictors scores when training–testing domain sequence similarity is < 0.7 (*p*-value < 0.029). The mean AUC scores for structure-based (blue) and sequence-based (magenta) predictors are plotted against sequence similarity threshold (bottom left and right).

In previous work, we showed that the performance of the sequence-based predictor depends on how similar in binding site sequence a given testing domain is to its nearest training domain. In particular, as the domain binding site sequence similarity decreases so does the predictor’s average performance until it is comparable to that of a naïve nearest neighbour sequence predictor [10]. To more rigorously compare structure-based and sequence-based predictor performance as training–testing domain sequence similarity varies, we performed a leave 12% of domains out cross validation with domain sequence similarity-based training set filtering for each predictor. For each fold, 12% of domains and their interactions were held out, and of the remaining domains, only those and their corresponding interactions were retained for training if the domain sequence similarity was less than a given threshold for all testing domains. All training sets had no more than 500 interactions. Ten folds were executed and repeated ten times for a total of 100 runs. For each run, the ROC and PR AUCs were computed and plotted as box plots according to the similarity threshold (Figure 3). A one-tailed t-test showed that the mean ROC and PR AUC scores were significantly higher for the structure-based predictor when training–testing domain sequence similarity is < 0.7 (*p*-value < 0.029). These results show that on average, the structure-based predictor is less dependent on training–testing domain sequence similarity compared to the sequence-based predictor at lower similarity thresholds.

### Structure-based predictions are validated by known PDZ domain-peptide interactions

We used the predictor to scan the human C-terminal proteome (defined by genome assembly Ensembl:GRCh37.64) [43] for binders of 45 PDZ domains with known interactions in PDZBase that we could obtain structures and compute features for. For each domain, this involved scanning 43827 unique C-termini of length five (including splice variants). Structures for these domains were obtained from the PDB or were homology modelled and are at least 35% sequence similar (average over 80%) to their template structures. The minimum QMEAN score for these models is 0.36 (average 0.78). Please see Additional file 2: Table S3 for more details.

The structure-based predictor has a true positive rate (TPR) of 0.36 and precision of 0.0033 and correctly predicted interactions for 22 of the 45 domains. For these domains approximately 73% of known PDZ domain-peptide interactions in PDZBase, an independent data source not used for training, were predicted (see Additional file 2: Table S4). The sequence-based predictor had a higher TPR of 0.46 and correctly predicted interactions for 28 out of 45 domains. For these domains, 65% of known PDZ interactions were predicted and the precision was 0.0024. Although the sequence-based predictor has a higher TPR than the structure-based predictor, its precision and coverage of known PDZ domains is lower. This is likely because the sequence-based predictor predicts on average more interactions per domains than the structure-based predictor (average 426.89 and 239.71 per domain respectively). The low precision for both predictors is due to the few known interactions per domain that are available from PDZBase (average 2.2 interactions per domain).

We also tested the false positive rate (FPR) of the predictor using two real negative data sets for human, which were used in a recent study [44] to benchmark a sequence-based predictor developed by Chen et al. [9]. The first data set consists of 466 experimentally validated negative interactions involving peptides that contain a PDZ binding motif found from the literature. The second data set consists of 133 negative literature-described interactions involving peptides with a non-binding PDZ motif caused by a mutation. The structure-based predictor made predictions for 410 negative interactions from the first data set and 126 negative interactions from the second data set, which resulted in an FPR of 0.145 and 0.0, respectively. The sequence-based predictor had a FPR of 0.09 and 0.0, and made predictions for 421 and 128 negative interactions for the first and second data sets, respectively. Compared to our structure-based and sequence-based predictors, the Chen sequence-based predictor has a much higher FPR of 0.482 and 0.256 for the first and second data sets, respectively [44] (see Additional file 2: Table S5).

### Many structure-based predictions correspond to known PDZ domain containing protein-protein interactions

To determine how many structure-based predicted interactions correspond to known PPIs, we scanned the human proteome to predict interactions for 218 human PDZ domains with known PPIs (that we could obtain structures and compute structure features for). Known PPIs were retrieved from iRefIndex [33], which is a database integrating interactions from different databases including BIND [45], BioGRID [46], CORUM [47], DIP [48], HPRD [49], IntAct [50] and MINT [51]. In total, 61 XRAY and nine NMR structures (only the first models used) were obtained from the PDB and 148 homology models were created. All models had a template sequence similarity of at least 22% (average 72%) and QMEAN score of at least 0.36 (average 0.78) Please see Additional file 2: Table S3 for more details.

In total, 88 domains had predicted interactions that corresponded to known PPIs, with an average of greater than 21% of known PPIs being correctly predicted per domain. The number of PPIs successfully predicted per domain was significant (*p*-value < 0.05, Fisher’s exact test) for all but ten domains. A caveat of this result is that PDZ domain containing proteins may contain multiple PDZ domains and other domains, so it is not possible to uniquely assign a PPI to a PDZ domain. This could result in erroneous false negative or true positive statistics for the above tests. However, the results still serve as an estimate of predictor performance and show that the predictor is able to predict many known human PPIs.

### The structure-based predictor is complementary to the sequence-based predictor

**Figure 4:**
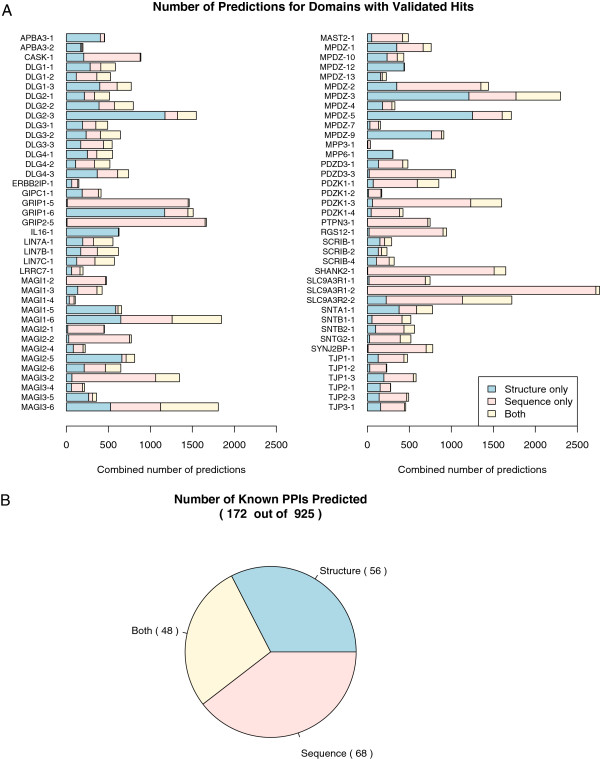
**Summary of predictions for domains with hits validated by known PPIs.** (**A**) Breakdown of the number of proteome scanning predictions per domain made by the structure-based predictor only (blue), sequence-based predictor only (pink), and both predictors (yellow). Only domains with hits matching known PPIs (physical and experimental interactions) in iRefIndex are shown. (**B**) Pie chart of the number of validated hits predicted by the structure-based predictor only (blue), sequence-based predictor only (pink), both predictors (yellow).

We next compared the structure-based predictor’s proteome scanning predictions to the ones obtained using our previously published sequence-based predictor [10]. In total, the results for 221 domains where both predictors were able to make predictions were compared. A total of 172 out of 925 known PPIs were predicted by both methods, 116 were unique to the sequence predictor and 56 were unique to the structure-based predictor (Figure 4). Thus the sequence and structure-based predictors both predict unique known PPIs and are complementary.

To better understand how unique predictions are made, we compared the results in more detail. The unique structure based predictions arise for different reasons. Some domains (43 domains) are more challenging for the sequence-based predictor, which returns a low number of hits per domain (ten or less) with none corresponding to known PPIs (see Additional file 2: Table S8) (e.g. APBA1-1, CNKSR2-1, IL16-1, IL16-3). The structure predictor fares better for nine of these domains (ARHGEF11-1, IL16-1, IL16-3, MPDZ-12, MPP6-1, PDZD2-3, PDZD2-5, RAPGEF6-1, SCRIB-3) and is able to predict many more hits per domain (on average approximately 510 hits) with on average approximately three known hits per domain. On the other hand, the structure-based predictor has difficulty predicting hits for 19 domains (e.g. DLG5-3, MPDZ-6, MPDZ-8), of which four are better predicted by the sequence-based predictor (MLLT4-1, MPDZ-8, MPP3-1, PDZD2-2; average 383 hits) with on average one known PPI hit per domain. In another scenario, two domains may have identical binding sites at the sequence level (e.g. DLG1-1 and DLG2-1), but be different at the structure level. The sequence-based predictor cannot distinguish between the two domains in this case, even though the domains may actually bind different proteins. While the structure-based predictor uses features corresponding to ten core positions, these features are computed by considering the entire domain structure. Therefore, even if two domains have the same binding site residues, the resulting features will be different if their whole domain structures are different. The structure-based predictor’s ability to distinguish between domains with highly similar binding site sequences helps explain why it is able to predict more unique interactions than the sequence-based predictor. Overall, these results demonstrate situations where the structure-based predictor can be used to make predictions for domains that otherwise could not be easily predicted by the sequence-based predictor and thus shows that both methods are complementary.

### Structure-based predicted binding specificities recapitulate experimental binding specificities

**Figure 5:**
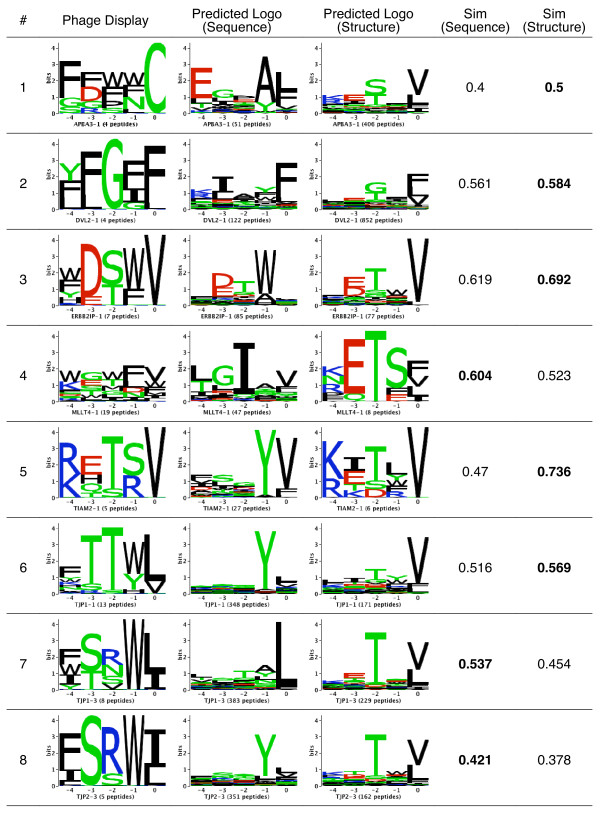
**Comparison of predicted and phage display determined binding specificities.** A comparison of phage display determined and predicted PDZ domain binding specificities for the last five terminal binding positions visualized as sequence logos. The binding specificity similarity between two domains was computed using the normalized Euclidean distance between their corresponding position weight matrices (see Additional file 1, equation 1). Non-genomic phage display peptides were removed from the set of binders for each domain. Only domains with four or more peptides after this filter were used to create sequence logos describing the domain’s binding specificity. Based on a previously established protocol, a peptide was considered to be genomic if the last four residues could be found in a proteomic tail, otherwise it was considered to be non genomic [10]. Numbers in bold indicate which similarity (sequence or structure) is higher (i.e. which predicted logo is closer to the experimental logo).

Since validation data is limited, we more generally assessed the results of proteome scanning by comparing predicted binding specificities to those known from phage display. We constructed position weight matrices to summarize the domain’s amino acid binding preference at each position in the ligand, using all predicted interacting peptides from C-terminal proteome scanning. Sequence logos were then used to visually represent the binding specificities. In total, 26 domains could be compared (i.e. they had less than four genomic peptides from phage display experiments), covering known PDZ domain binding classes I and II (see Additional file 1: Figure S3). For 14 domains, the structure-based predicted binding specificity is more similar to the phage display determined binding specificity than the sequence-based predicted binding specificity, and better recapitulates the preference of residues at specific positions. For example, the structure-based method better predicts the preference for hydrophobic residue Val at position 0 for ERBB2IP-1, for hydrophilic residues such as Gly or Thr at position −2 for DVL2-1 and for polar residues at position −4 and a Thr or Ser at position −1 for TIAM2-1 (position numbering counted backwards from the zero C-terminal position) (Figure 5). Three domains, APBA3-1, TJP1-3 and TJP2-3, had both structure-based and sequence-based predicted binding specificity similarities much lower than the average (less than 0.5). This seems to be caused by poor representation of these domains in the training set (Figure 5). More validation data should be used to more reliably compare the binding specificities for these domains in the future. Furthermore, since phage display experiments select optimal binders and cellular interactions may not be optimal (e.g. to aid interaction regulation), we expect some differences between phage display and proteome scanning-based profiles. In general, the similarity between the structure-based predicted and experimentally determined binding specificities is high (0.636).

### Predicted binding specificities are supported by known structural determinants of PDZ domain binding specificity

As noted above, there are many cases where the structure-based predicted binding specificity is closer to the experimental binding specificity than the sequence-based predicted binding specificity. For some examples, the structure-based predicted binding specificity better predicts the experimental binding specificity at certain positions (e.g. MLLT4-1, TJP1-1 and DVL2-1). To examine if this is caused by specific structural features used by the structure-based predictor, we searched the literature to find known structure determinants influencing these specific amino acid preferences and compared them to our results. For MLLT4-1, the structure-based predictions indicate a preference for a hydrophilic Thr residue at position −2. The preference for a hydrophilic Thr residue at position −2 is explained by the findings of Chen et al. [15]. Their work showed that the Thr preference at position −2 is due to its interaction with Gln at position α2-1 of the domain, which forms a hydrophilic binding site pocket at position −2. This preference is reflected in the structure-based predicted binding specificity, whereas a completely different preference for a hydrophobic Ile residue at this position is predicted by the sequence-based predictor (Figure 5). The domain TJP1-1 is another example where the predicted structure and sequence-based binding specificities are very different (Figure 5). Appleton et al., showed that this domain has a bi-specific preference for Trp or Tyr at position −1 [13]. The Trp preference is accommodated through main chain interactions with β2 and β3 strands, while the Tyr preference is accomplished through hydrogen bonding with Asp at position β3-5 of the domain. The bi-specific preference for a Trp or Tyr at position −1 is reflected in the structure-based binding specificity, while only a preference for Tyr is indicated in the sequence-based binding specificity. Finally, the predicted binding specificities for domain DVL2-1 are very different (Figure 5). Zhang et al. found that the −2 binding site of the domain actually accommodates a Gly-Tyr pair [52]. The preference for a Gly at position −2 is reflected in the predicted structure-based binding specificity whereas there is no obvious preference in the predicted sequence-based binding specificity. Since the binding specificities for these examples are determined by specific domain structure features, this helps explain why the structure-based predictor can better predict their binding preferences than the sequence-based predictor.

### A functional map of PDZ domain biology highlights PDZ involvement in a variety of biological processes

**Figure 6:**
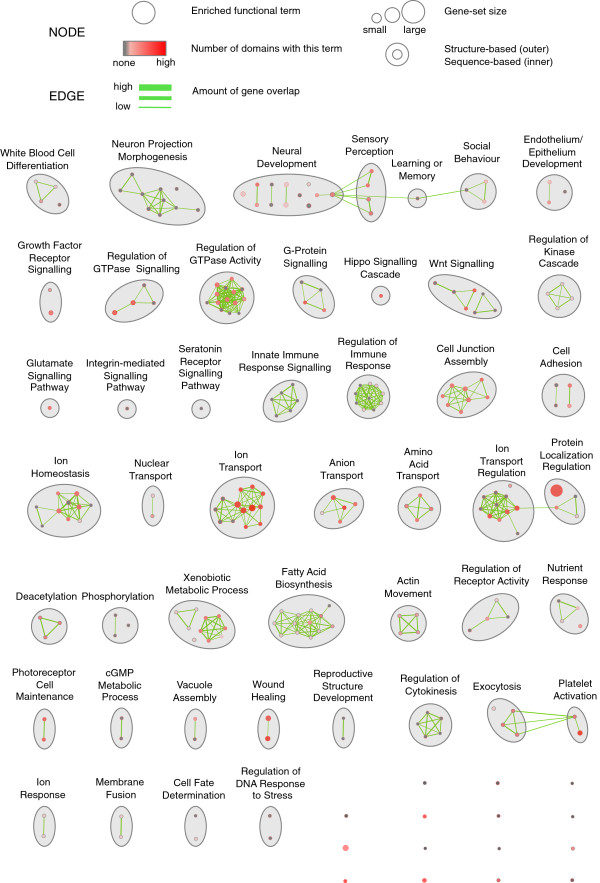
**A functional map of PDZ domain biology.** An enrichment analysis of the GO biological process terms associated with the predicted gene interactors for each of the domains from structure-based and sequence-based human proteome scanning was performed. The results were visualized as a network where the nodes represent gene-sets. The colour of the node border represents the number of domains that the gene-set was seen enriched for, among the structure-based predictions. The colour of the node centre represents number of domains that the gene-set was seen enriched for, among the sequence-based predictions. Edges represent the overlap between two connected gene-sets with the thickness corresponding to the number of genes overlapping. The complete enrichment map can be downloaded for interactive viewing in Cytoscape from http://www.baderlab.org/Data/StructurePDZProteomeScanning.

To identify gene functions better predicted by sequence or structure-based methods, we performed GO-based gene function enrichment analysis on all predicted hits. The results were visualized using an enrichment map, which groups related gene function terms to ease identification of functional themes (Figure 6). Enrichment results from both sequence and structure-based predictions were plotted on the same map to ease identification of overlapping or unique themes, with sequence-based enrichment scores corresponding to node centre colour and structure-based scores corresponding to node border colour. For example, a number of themes are enriched in hits from both methods, such as ‘photoreceptor cell maintenance’, ‘hippo signalling’ and ‘cell junction assembly’ (i.e. node centre and border are red). Other themes are only enriched in sequence-based (i.e. border is grey, node centre is red) or structure-based predictions (i.e. border is red, node centre is grey). For example, ‘neuron projection morphogenesis’, ‘regulation of cytokinesis’, and ‘innate immune response signalling’ themes contain terms only enriched in structure-based predictions, while ‘actin movement’, ‘membrane fusion’ and ‘nuclear transport’ are enriched only in sequence-based predictions.

**Figure 7:**
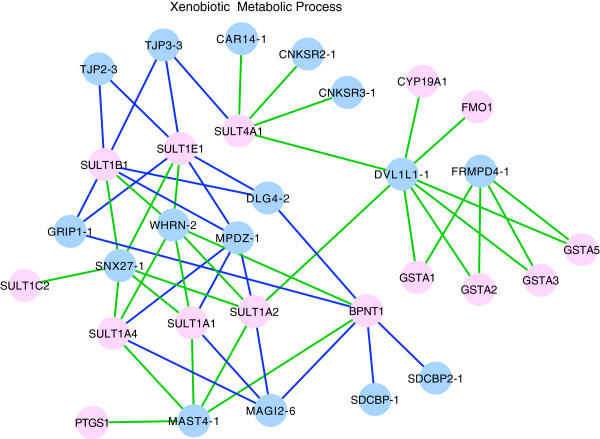
**A network view of predicted novel PDZ interactions in xenobiotic metabolism.** PDZ domains are shown as blue nodes and labelled using their gene names. Protein interactors are shown as pink nodes and labelled using their HGNC gene symbols. Blue edges represent structure-based only predicted interactions. Green edges represent sequence-based only predicted interactions. Only interactions involving proteins with GO annotations are presented.

We also compared the themes from our predictions to those from 1249 known PDZ mediated PPIs in the iRefIndex database [53]. Some themes were enriched only in known interactions (e.g. ‘DNA damage checkpoint’, ‘negative regulation of angiogenesis’), however many known themes were covered by our predictors (e.g. ‘cell junction assembly’, ‘ion homeostasis’, ‘neural development’). We identified the theme ‘xenobiotic metabolic process’ (enriched in both sequence-based and structure-based predictions) to be novel as it did not correspond to any themes seen in the known interaction network and did not have any PDZ interactions reported in the literature (based on a manual search). For this theme, both predictors predicted PDZ domain interaction with enzymes that are important for catalyzing foreign compounds in the xenobiotic metabolism pathway. For example the sequence-based predictor predicted the domain DVL1L1-1 to interact with cytochrome P450 (HGNC:CYP19A1) and dimethylaniline monooxygenase (HGNC:FMO1) [54,55], FRMPD4-1 to interact with various glutathione S-transferases (e.g. HGNC:GSTA1, GSTA2, GSTA3), MAST4-1 to interact with prostaglandin G/H synthase (HGNC:PTGS1). The domains SDCBP-1, SDCBP2-1 were predicted by the structure-based predictor to interact with bisphosphate nucleotidase (HGNC:BPNT1). The domains CAR14-1, CNKRS2-1, CNKRS3-1, SNX27-1, WHRN2-1 and the domains DLG4-2, GRIP1-1, MAGI2-6, MPDZ-1, TJP2-3 and TJP3-3 were predicted by the sequence-based and structure-based predictors respectively to interact with various sulfotransferases (e.g. HGNC:SULT1C2, SULT4A1, SULT1B1, SULT1E1, SULT1A1, SULT1A2, SULT1A4) (Figure 7 and Additional file 2: Tables S9-10).

In some cases, although the themes were also enriched in the iRefIndex map, only limited information about PDZ domain involvement in the associated process was found in the literature. These themes represent opportunities for our predictions to shed light on the role of PDZ domains where little is currently known. One example is ‘wound healing’, where both predictors predicted PDZ domains to interact with proteins involved in different stages of wound healing. These included platelet activators and aggregators (e.g. HGNC:CD9 [56], P2RY12 [57]), growth factor receptors (e.g. HGNC:PDGFRA [58], TGFBR1 [59], HGF [60]), plasma membrane calcium-transporting ATPases (e.g. HGNC:ATP2B1, ATP2B2, ATP2B3, ATP2B4 [61]), calcium-activated potassium channels (e.g. HGNC:KCNMA1, KCNMB2 [62]), fibrinogen (HGNC:FGG) [63], coagulation factors (e.g. HGNC:F8, F11 [64]), immune system proteins such as chemokines (e.g. HGNC:CXCR1, CXCR2, CCL19 [65]), tumour necrosis factors (e.g. HGNC:TNFAIP6, TNF [66]) and inhibitor of nuclear factor kappa-β kinase (HGNC:IKBKB) [66]) (Additional file 2: Tables S9-10).

Finally, our predictions also suggested additional interactions for well studied processes that are known to involve PDZ domains. For ‘Wnt signalling’, both predictors predicted known interactions between the domain MAGI3-2 and frizzled-4 and 7 as well as domains DLG4-1,2 and frizzled-1,2,4 and 7 [67]. However, several other PDZ domains were also predicted to interact with frizzled family members. Some examples include AHNAK2-1, CAR14-1, CNKSR2-1 (structure-based) and MPDZ-13, PDZRN4-1, SYNJ2BP-1 (sequence-based) which are all predicted to interact with one or more frizzled family members (HGNC:FZD1, FZD2, FZD4, FZD7, FZD10). Interactions which may negatively regulate Wnt signalling were also predicted and involve F-box-like proteins (HGNC:TBL1X, TBL1XR1) [68] and human colorectal mutant cancer protein (HGNC:MCC) [69] (Additional file 2: Tables S9-10).

Many functional themes we identify consist of multiple different enriched terms containing multiple proteins, predicted to interact with several PDZ domains. These patterns involve many proteins and are unlikely to occur by chance. Thus, our functional analysis provides additional validation of our prediction methods and highlights novel PDZ interactors involved in a variety of biological processes.

## Discussion

We have presented a structure-based predictor of PDZ domain-peptide interactions that can be used to scan C-terminal proteomes to predict PDZ domain mediated PPIs. Our predictor utilizes domain structure features derived from the whole domain, focusing on a core peptide-binding site defined by ten highly conserved amino acid positions. Combined with our use of experimentally determined and computationally generated training negative interactions, our predictor achieves high cross validation results and is expected to generalize well to unseen interactions in practice. Compared to our previous sequence-based predictor, the structure-based predictor is less dependent on training–testing domain sequence similarity and predicts many new validated interactions in human. As a result, the structure-based predictor is complementary to the sequence-based predictor and both should be used to identify candidates for further biological experiments and to expand our knowledge of PDZ domain mediated PPIs.

An important technical result of our work is our use of computationally generated negatives to supplement training and reduce over-prediction. We showed that the negative interactions in current experimental data sets do not adequately cover the negative proteome space resulting in a predictor that returns many hits that are likely false positives. While this problem is more apparent for the structure-based predictor, it also affects our sequence-based predictor, as there are several domains where sequence-based proteome scanning predicts thousands of hits, and likely affects other sequence-based predictors [10]. Since additional experimentally determined negatives for training are limited, using computationally generated negatives is required. While PWMs can be used to computationally generate such negatives as previously shown [10], such methods do not model dependencies between ligand positions and depend on a user or naively defined cutoff to discriminate between positives and negatives. Here, we use a semi supervised learning approach utilizing an SVM to generate additional negatives, since SVMs can better address the limitations faced by PWMs. As a result, the proteome scanning performance was improved by reducing the number of false positive hits that would otherwise be returned. As this problem is not unique to the structure-based predictor, training with additional negatives is likely to benefit other predictors as well.

Comparing proteome scanning hits to known PPIs, there is only a moderate overlap in hits predicted by both the structure-based and sequence-based predictor. While this suggests that the predictors are complementary and thus should both be used, there are cases when using either the structure-based or sequence-based predictor to find interactors may be more appropriate. For example, when the training–testing domain sequence similarity is < 0.7, the structure-based predictor may be more useful, since its performance is less dependent on sequence similarity at lower similarity levels. In fact, when the sequence similarity is very low the sequence-based predictor may fail to return any predictions. For other domains, a reliable structure may not be obtained or modelled, or the required structure features cannot be successfully generated. In this case, the sequence-based predictor may be the only predictor that can be used. However, for the majority of cases, both predictors should be used to find as many hits as possible for a given domain.

Although PDZ domains can recognize motifs internal to a protein, most data is available for domain-C-terminal binding, thus our predictors have been trained using this data and are best suited for the prediction of such interactions. Although other similar methods exist that are also available on the web, they can only predict that a protein containing a PDZ domain interacts with another protein [70] or are best suited for interactions between PDZ domains and specific types of proteins (e.g. membrane proteins) [71]. Thus, we expect our website will be useful to biologists in helping to further map the many processes mediated by PDZ domains.

While the current structure-based predictor performs well, other domain structure related features should be considered in the future. For example, it is known that the structural flexibility of the PDZ domain binding pocket can contribute to the domain’s ability to bind specific ligands [15,52]. Recently, a model of PDZ domain backbone flexibility was used to successfully predict domain binding specificity, but for a subset of human PDZ domains [19]. Thus, domain backbone flexibility features should be considered as they may help to improve predictor performance. Another structure related feature, which should also be considered, is binding pocket geometry and shape. Although we explored the use of 3D-Zernike descriptors [72], we found that their use did not benefit our predictor. However, there are other shape descriptors such as real spherical harmonic coefficients that could be investigated that may improve predictor performance [73]. Although we have built an entirely structure-based predictor, additional features including sequence features can be combined to build a single predictor that utilizes all available types of information. Finally, since the predictor predicts *in vitro* interactions, incorporating contextual information such as co-expression and protein location will help to build a more physiologically relevant map of PDZ domain mediated protein-protein interactions.

## Conclusions

We have presented a structure-based predictor of PDZ domain-peptide interactions using domain structure and peptide sequence information. Our predictor achieves high cross validation results and finds many interactions corresponding to known PDZ mediated PPIs not previously found by our sequence-based predictor. Using both predictors we defined a functional map of PDZ domain biology and identified novel PDZ interactors involved in a variety of biological processes. As a result, our predictions will help expand the coverage of current PDZ mediated PPI networks and provide new insight into the molecular mechanisms underlying a variety of biological processes.

## Availability and Requirements

For web-based proteome scanning:

Project name: POW! PDZ domain-peptide interaction prediction website

Project home page: http://webservice.baderlab.org/domains/POW/

Operating systems: Platform independent (web-based)

License: None

For proteome scanning software:

Project name: PDZ Structure-based Proteome Scanning

Project home page: http://baderlab.org/Data/StructurePDZProteomeScanning

Operating systems: Platform independent

Programming language: Java 1.5

License: Source code is freely available under the GNU Lesser Public General License (LPGL)

## Competing interests

The authors declare that they have no competing interests.

## Authors’ contributions

SH collected the data, developed and implemented the algorithms, drafted and revised this manuscript, and helped to develop the website. XX developed the website. GDB substantially contributed to all aspects of this project including conception and design, critically revised the manuscript and provided feedback about the website. All authors read and approved the final manuscript.

## Supplementary Material

Additional file 1 Supplementary Information. Click here for file

Additional file 2: Table S1 **Training domain structure information.** In total, 83 PDZ domains were used for training. Domain structures were obtained from the PDB or homology modelled through the Protein Model Portal. For NMR structures, only the first model was used. All homology models were generated by SWISS-MODEL and have greater than 50% sequence similarity to their template structure (average 90%). Model quality is estimated using template sequence ID (percentage of residues between target and template sequences that are identical) and QMEAN score (a scoring function that measures multiple geometrical aspects of protein structure, ranging from 0 to 1 with higher values indicating more reliable models). **Table S2. Blind test domain structure information.** Blind testing was performed using interaction data from mouse, worm and fly protein microarray experiments. In total, 13 mouse orphan, 7 worm and 6 fly PDZ domains were used. Homology models were generated by SWISS-MODEL. All models have at least 40% sequence identity to their template structures. An NMR structure was available for one fly domain and the first model was used. The average template sequence similarity was 0.92, 0.61 and 0.61 for mouse, worm and fly domains, respectively. One mouse domain (CHAPSYN-110-1) was removed from the test set because its performance was consistently poor for both predictors. Model quality is estimated using template sequence ID (percentage of residues between target and template sequences that are identical) and QMEAN score (a scoring function that measures multiple geometrical aspects of protein structure, ranging from 0 to 1 with higher values indicating more reliable models). **Table S3. Human proteome scanning domain structure information.** Proteome scanning was performed for 218 human PDZ domains, which have known interactions in iRefIndex. In total, 61 X-ray and nine NMR structures (only the first models used) were obtained from the PDB and 148 homology models were created (template sequence similarity minimum 22%, average 72%). Model quality is estimated using template sequence ID (percentage of residues between target and template sequences that are identical) and QMEAN score (a scoring function that measures multiple geometrical aspects of protein structure, ranging from 0 to 1 with higher values indicating more reliable models). **Table S4. Validation of structure-based predictions against known human PDZ domain-peptide interactions.** Proteome scanning predictions for 45 human PDZ domains were validated against known PDZ domain-peptide interactions in PDZBase. Several statistics were calculated including: # Positives, # TP (total number of true positives), # Predicted Structure (number of predictions predicted only by the structure-based predictor). # Predicted Sequence (number of predictions predicted only by the sequence-based predictor), # Predicted Both (number of predictions predicted by both), # TP Structure (number of true positives predicted by the structure-based predictor only), # TP Sequence (number of true positives predicted by the sequence-based predictor only), # TP Both (number of true positives predicted by both). **Table S5. Validation of structure-based predictions against known negative PDZ domain-peptide interactions for human. a. Negatives involving peptides with PDZ binding motifs.** Proteome scanning predictions for 74 human PDZ domains were validated against experimentally determined negative interactions involving peptides with PDZ binding motifs (found from the literature) for a total of 410 interactions. **b. Negatives involving peptides with non binding PDZ motifs.** Proteome scanning predictions for 24 human PDZ domains were validated against known negative interactions involving mutated peptides with non-binding PDZ motifs (found from the literature) for a total of 126 interactions. **Table S6. Validation of structure-based predictions against known experimentally determined PDZ domain-peptide interactions for worm**. Proteome scanning was performed for six worm PDZ domains with interactions from protein microarray experiments. Several statistics were calculated including the ones from Table S4 as well as the following: # Negatives, # FP Structure (number of false positives predicted by the structure-based predictor only), # FP Sequence (number of false positives predicted by the sequence-based predictor only), # FP Both (number of false positives predicted by both). **Table S7. Validation of structure-based predictions against known experimentally determined PDZ domain-peptide interactions for fly**. Proteome scanning was performed for seven fly PDZ domains with interactions from protein microarray experiments. Several statistics were calculated (see Table S6 caption). **Table S8. Validation of structure-based predictions against known protein-protein interactions**. Proteome scanning results for 221 human PDZ domains with both structure-based and sequence-based predictions were validated against known human PPIs in iRefIndex. A prediction is considered to be a true positive if the domain involved is found in a known PPI where one of the proteins contains the domain. See Table S4 caption for details about the calculated statistics. **Table S9. Structure-based predicted PDZ domain interactors for according to functional theme.** These tables contain domains, their sequence-based predicted interactors and the enriched functional theme (i.e. clusters in the Enrichment Map). **Table S10. Sequence-based predicted PDZ domain interactors according to functional theme.** These tables contain domains, their structure-based predicted interactors and the enriched functional theme (i.e. clusters in the Enrichment Map). Click here for file
