# Supplementary material for: Predicting PDZ domain mediated protein interactions from structure
Source: BMC Bioinformatics. 2013 Jan 21;14:27. doi: 10.1186/1471-2105-14-27 (PMC3602153; doi:10.1186/1471-2105-14-27)
Supplement: Additional file 1 — Supplementary Information. [file 1471-2105-14-27-S1.pdf]

Supplementary Information

Predicting PDZ Domain Mediated Protein Interactions from Structure

Shirley Hui[1,2], Xiang Xing[1,2], Gary D. Bader[1,2,3,§]

1 The Donnelly Centre, University of Toronto, Toronto ON, Canada

2 Department of Molecular Genetics, University of Toronto, Toronto ON, Canada

3 Department of Computer Science, University of Toronto, Toronto ON, Canada

§ Corresponding author

Email addresses:

SH: shirley.hui@utoronto.ca

XX: xiang.xing@utoronto.ca

GDB: gary.bader@utoronto.ca

Availability and Requirements

For website proteome scanning:

Project name: POW! PDZ domain peptide interaction prediction website

Project home page: http://webservice.baderlab.org/domains/POW/

Operating systems: Platform independent (web-based)

License: None

For proteome scanning software:

Project name: PDZ Structure-based Proteome Scanning

Project home page: http://baderlab.org/Data/StructurePDZProteomeScanning

Operating systems: Platform independent

Programming language: Java 1.5

License: Source code is freely available under the GNU Lesser Public General License (LPGL)

A. Parameters for structure feature generation software

1. Solvent accessibility and hydrogen bonding properties
    - Joy website [1]: http://tardis.nibio.go.jp/cgi-bin/joy/joy.cgi
    - PDB files were uploaded and the resulting LaTEX output file was downloaded and parsed.
2. Solvent accessible area
    - Surfv software [2]: http://wiki.c2b2.columbia.edu/honiglab_public/index.php/Software:SURFace_Algorithms
    - The software was run using the parameters: single format flag = on, resolution = 2, probe size = 1.4, last 3 parameters = 1, 0 and 0.
3. Electrostatic and hydrophobicity
    - VASCo software: http://genome.tugraz.at/VASCo
    - The software uses the the program DelPhi [3, 4] to compute the electrostatic potentials and HydroCalc to compute the hydrophobicity values. The default parameters, as distributed in the VASCo package for these programs, were used. Both programs require the calculation of surface points which as performed by the MSMS software [5]. For all programs the default probe size of 1.4 was used.
4. 3D binding pocket shape
    - 3D-Surfer website [6]: http://dragon.bio.purdue.edu/3d-surfer

- PDB coordinates corresponding to the binding pocket (defined by 10 core positions) were uploaded and the resulting Zernike descriptors were collected.

B. Binding specificity similarity calculation

The distance between two PWMs a and b is the normalized Euclidean distance:

$$\text{Distance}_{PWM}(a,b) = \frac{1}{\sqrt{2}} \sum_{i=1}^{n} \sqrt{\sum_{L \in (20aa's)} (a_{i,L}, b_{i,L})^2}$$

$$\text{Similarity}_{\text{PWM}}(a,b) = 1.0 - \text{Distance}_{\text{PWM}}(a,b) \qquad \text{equation 2}$$

where n is the number of columns in the PWM. This metric is normalized such that 0 represents perfectly similar PWMs and 1 represents perfectly dissimilar PWMs. The similarity between two PWMs is therefore 1 minus the distance.

C. Comparison of cross validation results predictors trained using 10 vs. 16-position domain binding site definition

Table S1. Comparison of cross validation results for predictors trained using a data set with domain features corresponding to a ten-position versus a 16-position domain binding site definition. The ten positions were defined by Tonikian et al. [7] and are based on positions that are in close contact with the peptide ligand (< 4.5 angstroms) across nine PDZ domain structures. The 16 positions were defined by Chen et al. [8] and were derived similarly from a single PDZ domain-ligand complex structure. Using the 16-position binding site definition, 556 positive and 1167 negative interactions corresponding to 58 domains were used for training. Four different cross validation strategies used. First we performed ten fold cross validation which involves partitioning the training data into ten randomly selected interaction sets, independently holding out each set for testing against a predictor trained using the remainder of the data, and

computing average performance across all ten runs. We then held out 12% of the domains (to estimate performance dependence on specific sets of domains), 8% of the peptides (to estimate predictor performance dependence on specific sets of peptides) and both 12% of the domains and 8% of the peptides (to estimate predictor performance dependence on specific sets of domains and peptides) and tested on the rest, again repeating this ten times. The cross validation AUC scores for the 10-position domain binding site definition are higher across all strategies. This indicates that the information in the smaller binding site definition is adequate to achieve good predictor results and it is not necessary to train with additional features from the 16 binding site positions. Since the ten positions are also based on multiple PDZ domain structures, these positions likely capture more general features about PDZ domain binding compared to the 16 positions which were derived from a single structure and may contain noise when applied to other PDZ domains. Finally, using the smallest number of features for training helps to prevent the predictor from becoming overfit and further justifies the use of the ten-position binding site definition.

	ROC AUC		PR AUC	
	10 positions	16 positions	10 positions	16 positions
10 Fold	**0.96**	0.936	**0.936**	0.894
Domain	**0.872**	0.840	**0.785**	0.708
Peptide	**0.935**	0.907	**0.909**	0.844
Domain+Peptide	**0.927**	0.925	**0.886**	0.878

D. Domain structure feature set hold out cross validation results

Figure S1. Cross validation results for structure-based predictors trained using different combinations of structure features. Initially, five types of structure features were

considered for feature encoding: Joy (solvent accessibility, hydrogen bonding) [1], Surfv (solvent accessible area) [2], VASCo (electrostatics), VASCo (hydrophobicity) [9] and 3D Zernike descriptors (structure shape) [6]. Five predictors were trained with all but one of the feature sets and the performance for multiple cross validation strategies was measured. For all strategies except for the leave 12% of domains out, the performance across all predictors is comparable. For the strategy that involved leaving sets of domains out, the performance improves only if the 3D Zernike descriptors are not used. Therefore, the final domain structure feature encoding did not include these features.

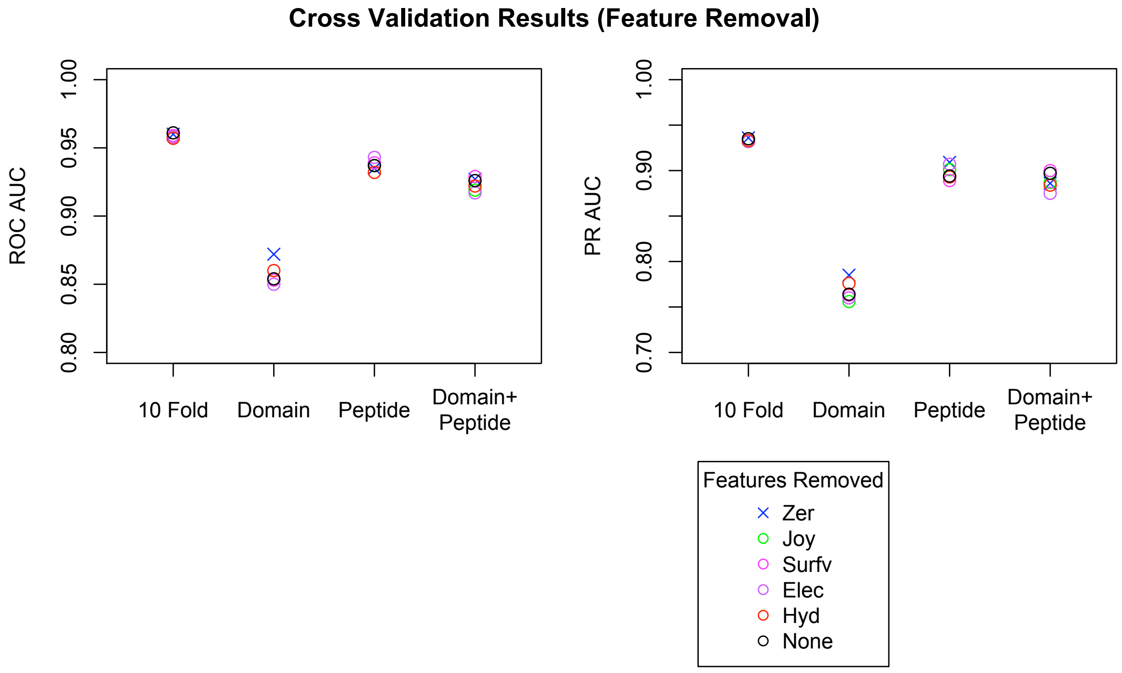

E. Semi-supervised negative training set expansion

An initial predictor was built using the data for 88 PDZ domains described above. A preliminary assessment of the predictor's proteome scanning performance was performed by scanning the human proteome (defined by genome assembly Ensembl:37.64) for binders for each domain in the training set. This test revealed that the predictor returned a

large number of hits (1000 or more) for over half of the domains. The average number of predictions returned per domain was over 2000 (see Figure S2, left boxplot). Since previous phage display experiments detected fewer than a hundred binders per domain among billions of random peptides, the majority of these initial predictions are likely false positives. We surmised that the initial negative training data did not adequately cover the negative proteomic interaction space. Therefore, we added additional negative interactions for the 24 human and 22 mouse domains that the predictor returned 1000 or more hits for.

We used a semi supervised learning approach similar to a method previously used to expand negative training data sets when there are no negatives initially available [10]. For the human domains, a SVM was trained using the initial human training data. This SVM was then used to predict additional negative interactors by scanning a pool of unlabelled non-redundant C-terminal peptides obtained from the human proteome (in total 2522 peptides). The negative interactors were then sorted in order by decreasing decision value and 100 peptides were sampled. The same was repeated using the initial training data for mouse with the pool of non-redundant mouse proteomic C-terminal peptides (in total 2348 peptides). A SVM for both mouse and human was then trained using all the initial training data plus the additional predicted negative interactions.

We used this predictor to scan the human proteome for interactors of training domains and found that fewer domains (16 out of 88 domain or 18%) still had 1000 or more predicted hits. For all but one domain, which had no change, the number of predictions

returned per domain was lower than before, with an average number of predictions returned per domain of approximately 685 (Figure S2, middle boxplot). However, for five domains, the predictor still predicted over 2000 interactions. We considered these to be outliers and removed these domains from the training set. We then repeated the above steps to train the final predictor using a total of 942 positive and 1843 negative interactions involving 83 PDZ domains and 872 peptides.

When scanning the human proteome again for only training domains, the final predictor predicted 1000 or more hits for only five out of 83 domains (approximately 6% of training domains). The average number of predictions per domain returned by the final predictor was approximately 400 (Figure S2, right boxplot). We did not remove any more domains from the training set to avoid removing too many positive interactions from the data set.

F. Number of hits returned during negative training set expansion

Figure S2. Boxplots of the number of hits returned by different structure-based predictors during negative training set expansion. Left boxplot. An initial predictor was built using all available training data corresponding to 88 PDZ domains. When proteome scanning was performed for only the training domains, the predictor returned a large number of hits (1000 or more) for over half of the domains. In general, the mean number of predictions returned per domain was over 2000. Middle boxplot. Additional negative training data was generated by using an SVM to scan a pool of proteomic human or mouse peptides. The resulting predictor predicted 1000 or more hits for 18% of training domains with a mean number of predictions returned per domain of 685. Right boxplot.

For five domains, the predictor still predicted over 2000 interactions and we considered these to be outliers and removed these domains from the training set. The final predictor uses training data for 83 PDZ domains. The average number of predictions per training domain returned by the final predictor was 406.

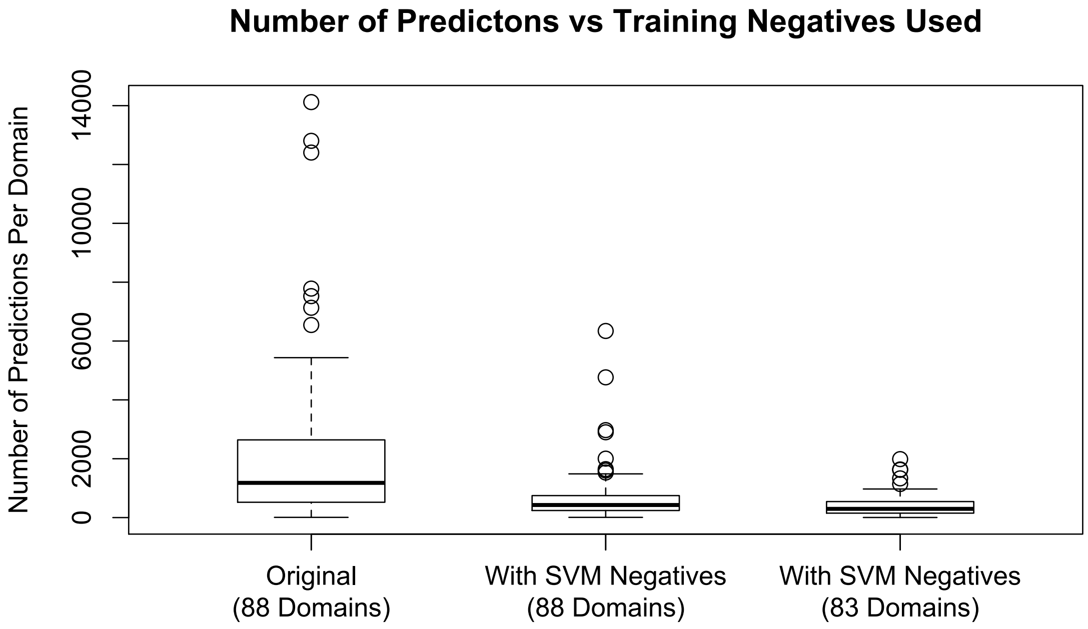

G. Comparison of predicted and experimentally determined genomic binding specificities for human PDZ domains

Figure S3. The predicted and phage display determined binding specificities for 26 human PDZ domains with four or more genomic peptides were visualized as sequence logos and compared. The binding specificity similarity between two domains was computed using the normalized Euclidean distance between their corresponding position weight matrices (See Section D). Non-genomic phage display peptides were removed from the set of binders for each domain. Based on a previously established protocol, a

peptide was considered to be genomic if the last four residues can be found in a proteomic tail, otherwise it was considered to be non genomic [11]. Numbers in bold indicate which similarity (sequence or structure) is higher (i.e. which predicted logo is closer to the experimental logo).

#	Phage Display	Predicted Logo (Sequence)	Predicted Logo (Structure)	Sim (Sequence)	Sim (Structure)
1	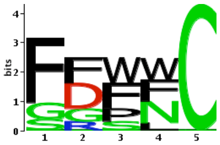 APBA3-1 (4 peptides)	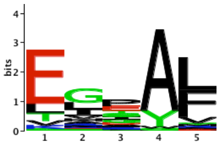 APBA3-1 (51 peptides)	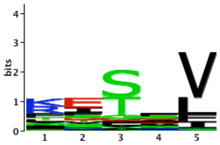 APBA3-1 (406 peptides)	0.4	**0.5**
2	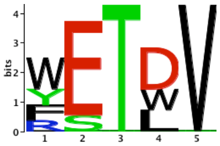 DLG1-1 (6 peptides)	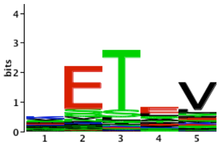 DLG1-1 (300 peptides)	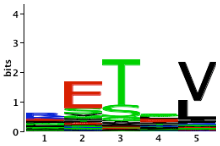 DLG1-1 (456 peptides)	**0.698**	0.673
3	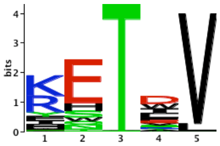 DLG1-2 (22 peptides)	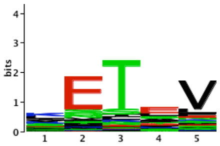 DLG1-2 (408 peptides)	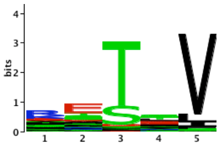 DLG1-2 (279 peptides)	0.781	**0.812**
4	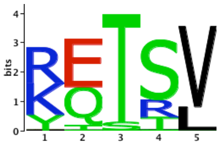 DLG1-3 (10 peptides)	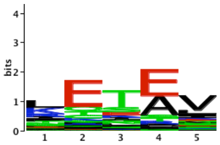 DLG1-3 (374 peptides)	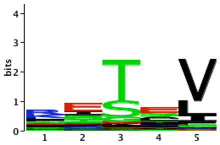 DLG1-3 (566 peptides)	0.634	**0.711**
5	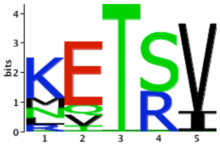 DLG2-3 (10 peptides)	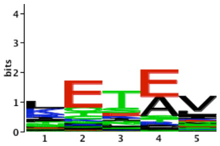 DLG2-3 (374 peptides)	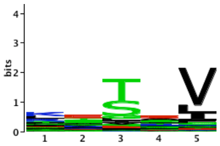 DLG2-3 (1399 peptides)	**0.642**	**0.642**
6	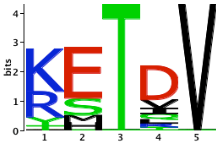 DLG3-2 (16 peptides)	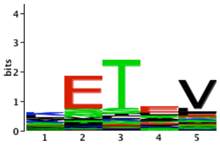 DLG3-2 (408 peptides)	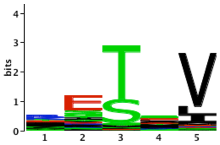 DLG3-2 (472 peptides)	**0.709**	**0.709**
7	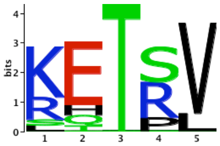 DLG4-3 (9 peptides)	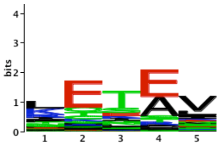 DLG4-3 (374 peptides)	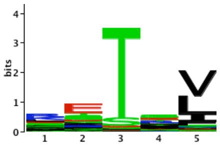 DLG4-3 (497 peptides)	0.647	**0.718**
8	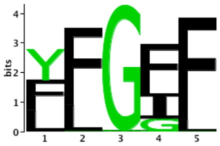 DVL2-1 (4 peptides)	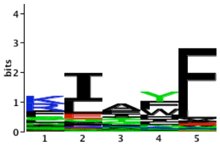 DVL2-1 (122 peptides)	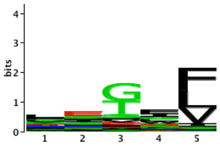 DVL2-1 (852 peptides)	0.561	**0.584**

9	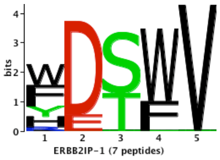	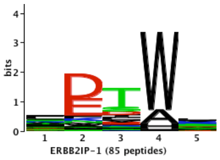	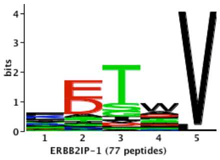	0.619	**0.692**
10	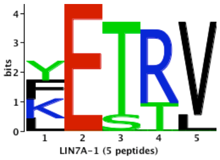	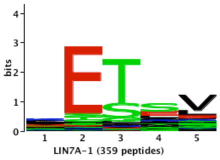	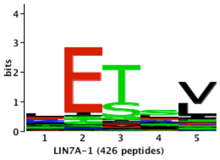	**0.701**	0.696
11	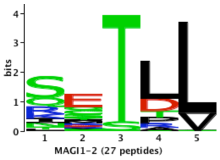	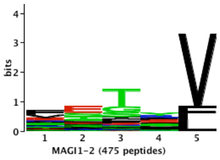	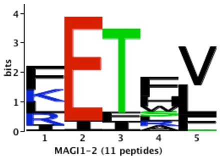	0.612	**0.627**
12	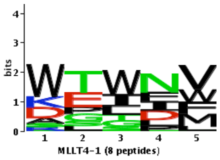	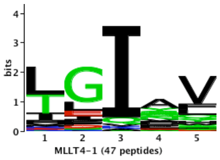	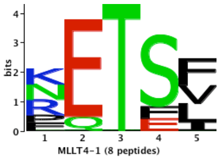	**0.604**	0.523
13	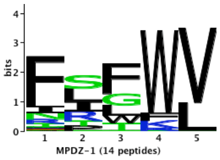	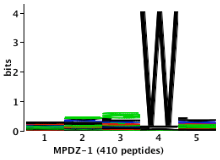	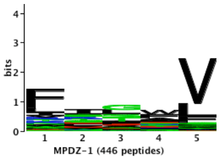	0.69	**0.746**
14	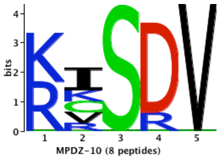	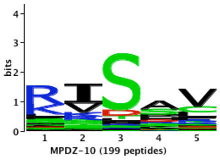	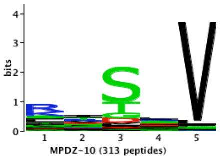	0.678	**0.691**
15	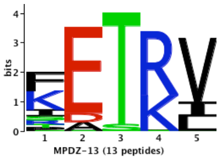	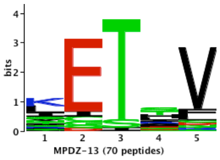	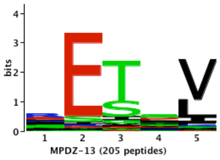	**0.777**	0.728
16	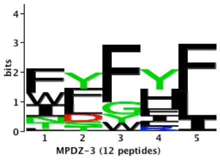	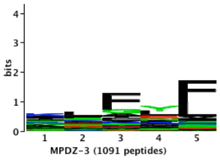	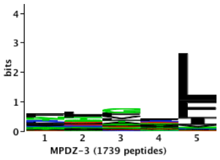	**0.781**	0.713

17	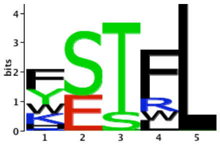 PDZK1-1 (13 peptides)	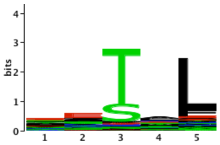 PDZK1-1 (781 peptides)	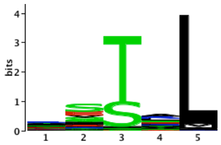 PDZK1-1 (326 peptides)	0.693	**0.757**
18	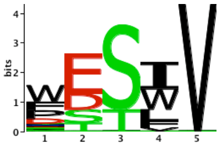 PTPN13-2 (14 peptides)	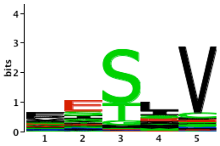 PTPN13-2 (222 peptides)	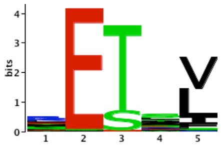 PTPN13-2 (108 peptides)	**0.793**	0.562
19	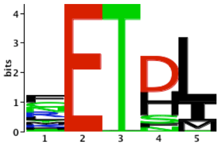 SCRIB-1 (23 peptides)	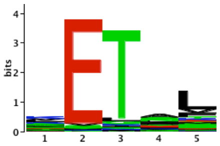 SCRIB-1 (139 peptides)	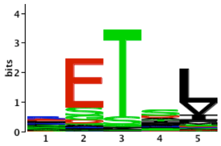 SCRIB-1 (234 peptides)	**0.796**	0.771
20	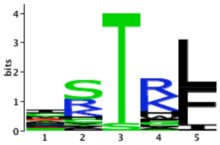 SHANK3-1 (35 peptides)	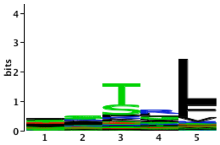 SHANK3-1 (1523 peptides)	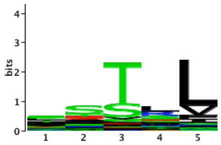 SHANK3-1 (864 peptides)	**0.771**	0.765
21	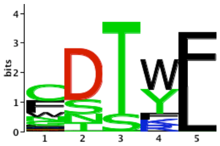 SLC9A3R2-2 (14 peptides)	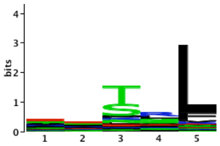 SLC9A3R2-2 (1496 peptides)	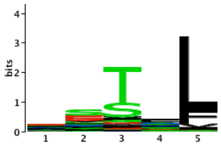 SLC9A3R2-2 (811 peptides)	0.621	**0.675**
22	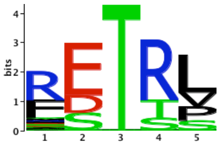 SNTA1-1 (7 peptides)	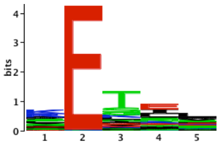 SNTA1-1 (398 peptides)	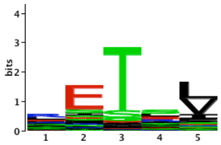 SNTA1-1 (568 peptides)	0.629	**0.741**
23	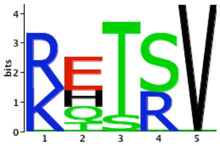 TIAM2-1 (5 peptides)	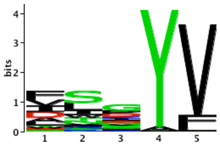 TIAM2-1 (27 peptides)	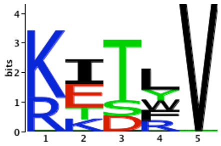 TIAM2-1 (6 peptides)	0.47	**0.736**
24	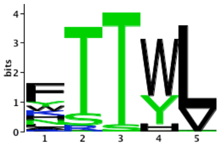 TJP1-1 (13 peptides)	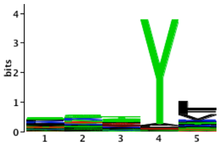 TJP1-1 (348 peptides)	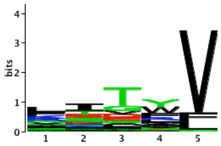 TJP1-1 (171 peptides)	0.516	**0.569**

25	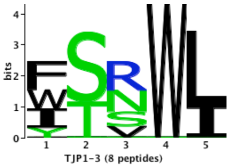	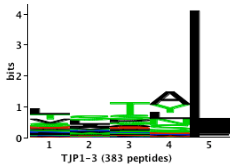	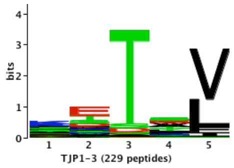	**0.537**	0.454
26	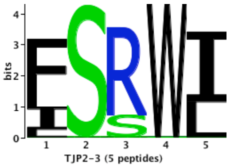	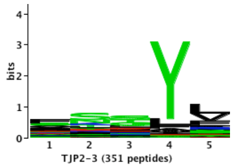	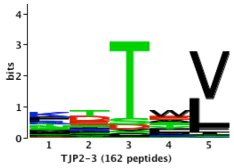	**0.421**	0.378

H. Structure based predictor blind testing performance

Blind testing was performed to obtain an unbiased measure of predictor performance and to determine if the predictor could correctly predict interactions in other organisms not represented in the training set (such as fly and worm). We used interaction data for 13 mouse, seven worm and six fly PDZ domains with interactions from previous protein microarray experiments which were not previously used for training [8] (Table S2). Homology models were generated by SWISS-MODEL and have at least 40% sequence identity to their template structures and no binding site gaps. The average template sequence similarity was 92%, 61% and 61% for mouse, worm and fly domains, respectively. An NMR structure was available for one fly domain (PAR6-1) and the first model was used (1RY4 A). One mouse domain (CHAPSYN-110-1) was removed from the test set because its performance was consistently poor for both sequence-based and structure-based predictors (see Additional file 2, Table S2).

The blind test results show that the structure-based predictor is able to correctly predict many unseen interactions in fly, worm and mouse (Figure S4) and that its performance is similar to the sequence-based predictor (Table S3), but somewhat worse for worm and

fly. Since only a few domains are tested in these test sets, additional data is required to accurately assess blind test performance.

Figure S4. Blind testing ROC and Precision/Recall curves for mouse (magenta), worm (green) and fly (black). Test data was obtained from published protein microarray experiments. Number of PDZ domains tested is noted in parentheses.

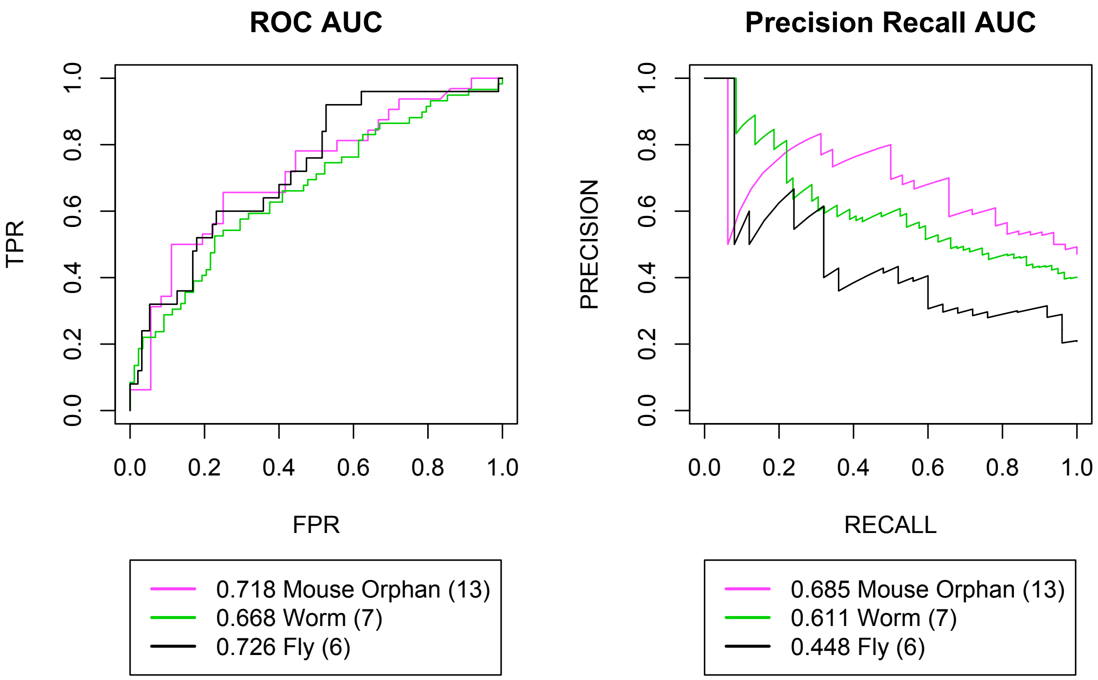

Table S2. Summary of data used for blind testing

		Domain		Interactions	
Organism	Source	# Pos	# Neg	# Pos	# Neg
Mouse (Orphan)	Protein microarray	8	13	32	36
Worm	Protein microarray	6	7	59	88
Fly	Protein microarray	6	6	34	106

Table S3. Structure-based and sequence-based predictor blind testing performance

	ROC AUC		PR AUC	
	Structure	Sequence	Structure	Sequence
Mouse (Orphan)	0.718	0.709	0.685	0.723

Worm	0.668	0.718	0.611	0.663
Fly	0.726	0.799	0.448	0.591

I. Proteome scanning results for fly and worm

We scanned the worm and fly proteomes (defined by genome assemblies Ensembl:WS220.64 and Ensembl:BDGP5.25.64) to predict binders for six worm and seven fly domains with experimentally determined positive and negative interactions and for which we could obtain structures and compute features for. This involved scanning 20167 and 14476 unique C termini of length five for worm and fly, respectively (including splice variants). Structures for the domains were obtained from the PDB or were homology modelled and are at least 40% sequence similar (average over 60%) to their template structures (see Additional file 2, Table S2, Table S3).

The predictor correctly predicted interactions for all worm (six out of six) and five out of seven fly PDZ domains, respectively. For these domains, approximately 46% and 54% of known interactions for worm and fly, respectively, were predicted (see Additional file 2, Table S6, Table S7). Using the negative interactions from the protein microarray experiments, we computed the FPR and found it to be on average 0.197 and 0.157 for worm and fly, respectively. These results suggest that the predictor is able to correctly predict both positive and negative PDZ domain-peptide interactions in different organisms.

Reference

1. Mizuguchi K, Deane CM, Blundell TL, Johnson MS, Overington JP: **JOY: protein sequence-structure representation and analysis.** *Bioinformatics* 1998, **14:**617-623.
2. Sridharan S, Nicholls A, Honig B: **A new vertex algorithm to calculate solvent accessible surface areas.** *J Biophys* 1992, **61:**A174.
3. Rocchia W, Alexov E, Honig B: **Extending the Applicability of the Nonlinear Poisson−Boltzmann Equation: Multiple Dielectric Constants and Multivalent Ions.** *J Phys Chem B* 2001, **105:**8.
4. Rocchia W, Sridharan S, Nicholls A, Alexov E, Chiabrera A, Honig B: **Rapid grid-based construction of the molecular surface and the use of induced surface charge to calculate reaction field energies: applications to the molecular systems and geometric objects.** *J Comput Chem* 2002, **23:**128-137.
5. Sanner MF, Olson AJ, Spehner JC: **Reduced surface: an efficient way to compute molecular surfaces.** *Biopolymers* 1996, **38:**305-320.
6. La D, Esquivel-Rodriguez J, Venkatraman V, Li B, Sael L, Ueng S, Ahrendt S, Kihara D: **3D-SURFER: software for high-throughput protein surface comparison and analysis.** *Bioinformatics* 2009, **25:**2843-2844.
7. Tonikian R, Zhang Y, Sazinsky SL, Currell B, Yeh JH, Reva B, Held HA, Appleton BA, Evangelista M, Wu Y, et al: **A specificity map for the PDZ domain family.** *PLoS Biol* 2008, **6:**e239.
8. Chen JR, Chang BH, Allen JE, Stiffler MA, MacBeath G: **Predicting PDZ domain-peptide interactions from primary sequences.** *Nat Biotechnol* 2008, **26:**1041-1045.
9. Steinkellner G, Rader R, Thallinger GG, Kratky C, Gruber K: **VASCo: computation and visualization of annotated protein surface contacts.** *BMC Bioinformatics* 2009, **10:**32.
10. Wang C, Ding C, Meraz RF, Holbrook SR: **PSoL: a positive sample only learning algorithm for finding non-coding RNA genes.** *Bioinformatics* 2006, **22:**2590-2596.
11. Hui S, Bader GD: **Proteome scanning to predict PDZ domain interactions using support vector machines.** *BMC Bioinformatics* 2010, **11:**507.
